# Introverted and yet effective? A faceted approach to the relationship between leadership and extraversion

**DOI:** 10.3389/fpsyg.2023.1185271

**Published:** 2023-08-10

**Authors:** Simon Liegl, Marco R. Furtner

**Affiliations:** ^1^Liechtenstein Business School, University of Liechtenstein, Vaduz, Liechtenstein; ^2^Department of Psychology, University of Innsbruck, Innsbruck, Austria

**Keywords:** leadership behaviors, full range leadership, extraversion, introversion, assertiveness, sociability, leadership effectiveness, leadership emergence

## Abstract

**Introduction:**

Extraversion and its facets of assertiveness and sociability were identified as stable predictors for leader emergence and effectiveness. However, recent research suggested that extraversion may lie in the eyes of the beholder; it might not be the leader’s possession but their followers’ attribution of the trait that shapes these criteria of leader success.

**Methods:**

In our study, we reverse-engineered this relationship and assessed the effects of effective leadership behaviors on personality perceptions. More specifically, we created scenarios of a leader responding to coordination challenges with passive-avoidant, transactional, or transformational leadership behaviors. We presented 204 participants with these scenarios and assessed how extraverted, assertive, and sociable they perceived the leader to be.

**Results:**

Interestingly, and not fully meeting our expectations, ascriptions of extraversion and its facets of assertiveness and sociability did not directly relate to the effectiveness of the behaviors, as the moderately effective transactional leadership style garnered the highest ascriptions of extraversion and its facets. Further, ascriptions of extraversion to the transformational behavior of intellectual stimulation were remarkably low, matched only by the laissez-faire dimension of the passive-avoidant leadership style.

**Discussion:**

We integrate and contrast these unexpected but explainable findings with current research, discuss potential associations between introversion and empowering leadership practices and provide suggestions for future discourse, illustrating the potential of investigating the presence of an introverted leadership advantage in the workplace of tomorrow.

## 1. Introduction

While many specific states and contexts affect our everyday experiences and behaviors at work, interindividual differences, first and foremost personality, were repeatedly identified as the most stable predictors of various workplace outcomes (e.g., Barrick and Mount, 1991; Furnham, 2008). Employees distinguish themselves through their conscientiousness, one of the best indicators of job performance (Wilmot and Ones, 2019), while the modern workplace appears to necessitate a high level of openness, as this trait facilitates solving complex problems and adapting to steadily and rapidly changing environments (D’Zurilla et al., 2011; Silvia and Christensen, 2020; Maran et al., 2022).

When it comes to leaders, extraversion was identified as the most consistent antecedent for their emergence and performance (Judge et al., 2002; Ensari et al., 2011; Zaccaro et al., 2018; Wilmot et al., 2019). However, while this so-called extraverted leadership advantage (Grant et al., 2011) is empirically well-funded, this previous research mostly focused on the relationship between extraversion as a characteristic of the leader and their leadership style and behaviors. We yet know little about how leadership styles and behaviors shape followers’ impressions of extraversion. Investigating the observers’ rather than the leaders’ point of view becomes increasingly relevant given recent research introducing the notion that the relationship between leaders’ extraversion and their success is mostly based on displayed and thus perceived state extraversion (Spark and O’Connor, 2021), indicating that this effect mostly lies in the eyes of the beholder. And while this study could show that acting extraverted, even when it’s counterdispositional to one’s preferred behavior, can exert similar effects on leader emergence than trait extraversion does, we firstly aim for a more direct investigation of how leader behaviors shape followers’ impressions, and secondly focus on the role of active and effective leadership practices. In this, we were guided by the questions of how new employees judge an incumbent leader, given that they are directly exposed to actual leadership behaviors rather than observing an aspiring leader and whether the association between extraversion and leader effectiveness is actually so profoundly ingrained within ourselves that it acts as a potential halo effect (i.e., the generalization of a single observed positive characteristic to other unobserved characteristics; Thorndike, 1920), and we implicitly assess effective leadership as a direct indicator for extraversion. To provide insights based on this shift from the entrenched perspective, we conducted an experimental investigation into the ascriptions individuals form about leaders based on descriptions of their leader behaviors alone. We presented participants with leadership scenarios accompanied by behavioral responses based on the full-range leadership model (Bass, 1985) to assess whether more active and effective leadership behaviors are associated with ratings of a more active, i.e., an extraverted, and more concretely assertive and sociable personality. Should the effectiveness of the displayed behaviors be in direct accordance with ascriptions of extraversion, this would place an even bigger emphasis on the role of state-extraversion and the potential necessity for leaders to engage in active and effective leadership behaviors in order to increase their chance of success, even if this is a counterdispositional behavior for them (McNiel and Fleeson, 2006; Fleeson and Gallagher, 2009; Spark and O’Connor, 2021).

## 2. Theoretical background and hypothesis development

### 2.1. Evolutionary leadership and signaling theory

Following the evolutionary leadership theory (van Vugt et al., 2008; van Vugt and Ronay, 2014), leadership constitutes a prerequisite for social groupings to advance, as it takes a leader to coordinate individuals and influence them to venture into a promising yet uncertain future together. However, for one individual to be able to wield such influence and power, they must first gather a followership that accepts their guidance (Grabo et al., 2017). According to the signaling theory (Spence, 1973), individuals achieve this by sending observable behaviors (i.e., signals) that indicate their intent and aptitude to coordinate others. In more detail, the signaling theory describes the process of reducing information asymmetry between two entities. The entity possessing information about their own characteristics or intentions, the other entity wishes to access, may act as a sender and employ signals that are designed to communicate this information (Spence, 1973). For a leader this might entail signaling their personality, intelligence, emotions, or motivation to lead (Connelly et al., 2011). These signals are then received and processed by the second entity (the receiver) to infer the desired information and send feedback (Stiglitz, 2000; Elitzur and Gavious, 2003). For a behavior to actually act as a signal and be deemed as an honest representation of the underlying characteristic it needs to be costly to produce, indicating that it requires less effort to be produced by entities possessing the characteristics than by entities that do not (Spence, 1973; Connelly et al., 2011). To assess the characteristics and qualifications of aspiring leaders, followers resort to an implicit and latent regulatory variable, the so-called *leader index* (Tooby and Cosmides, 2008; Grabo et al., 2017), that includes signals as well as further contextual and attributional factors and allows for rapid judgments of someone’s eligibility to become a leader (Spisak et al., 2012; Grabo et al., 2017). Ultimately, these should lead followers to collectively invest their efforts in the individual that possesses the most pronounced leader index and is therefore best equipped to coordinate the group to solve the challenge at hand thus causing them to emerge and be more effective (Spisak et al., 2012; Grabo et al., 2017).

### 2.2. Extraversion and leader emergence

Which are the signaled characteristics that inform followers’ selection decisions? The trait theory of leadership posits that certain intrapersonal traits predict leaders’ emergence and effectiveness (e.g., Judge et al., 2002). Meta-analytic results on the five-factor model of personality consistently identified extraversion as the paramount predictor for both of these criteria for leader success (Judge et al., 2002; Ensari et al., 2011; Zaccaro et al., 2018; Wilmot et al., 2019). Further, extraversion is not only closely related to transformational leadership (e.g., Bono and Judge, 2004), the most effective style within to the full-range leadership model (Bass, 1985), but also increases a leader’s likelihood of obtaining both informal and formal leadership roles (Spark et al., 2022). The overall positive and consistent relationships between extraversion and both leader emergence and performance led to the term “extraverted leadership advantage” (Grant et al., 2011) indicating that individuals high in extraversion possess an inherent predisposition for leadership success. However, these findings focus on the signaler within the signaling process, rather than the receivers’ reactions toward perceived signals. As leader emergence and ultimately effectiveness is based on behavioral signaling (Grabo et al., 2017), and being extraverted appears to lead to an increased tendency to show effective leader behaviors (Bono and Judge, 2004), thus granting a leadership advantage (Grant et al., 2011), the continuation of this effect on the receivers’ side, i.e., effective leadership behaviors leading to ascriptions of extraversion, should also be observable. This reverse link is supported by findings indicating a similarity bias in the perception of leaders (Felfe and Schyns, 2010). In fact, even acting extraverted without possessing the extraversion trait was shown to reproduce the extraverted leadership advantage (Spark and O’Connor, 2021), thus further positioning the extraverted leadership advantage in the hands of the receivers. Additionally, the actual effectiveness of leadership is fundamentally shaped by others’ subjective and thus potentially biased attributions thereof (e.g., Wong and Giessner, 2018). We therefore adopted the perspective of a state–trait-model of leadership (Spark and O’Connor, 2021) and focused on leader behaviors that can elicit desirable trait impressions in followers.

### 2.3. Leadership and personality

Our study seeks to provide insights into the reverse effect of leadership behaviors on extraversion ascriptions on two levels; first, by examining higher-order personality dimensions, and, second, by further dissecting their subordinate facets.

According to the full range model of leadership (Bass, 1985), transformational leadership, by definition, constitutes the upper echelon of leadership styles in terms of its effectiveness and can be related to various positive leadership outcomes (e.g., Zaccaro et al., 2018). This particular type of leadership is focused on leader proactivity and interactions between leaders and followers, which becomes more apparent when looking at its four constituents, namely idealized influence (i.e., being perceived and acting as a role model), inspirational motivation (providing an inspiring vision), intellectual stimulation (encouraging intellectual engagement), and individualized consideration (developing employee competencies; Bass, 1985). The traditional transactional leadership style is characterized by a mutual exchange of resources (contingent reward) and actively aiming to prevent problems and mistakes (active management by exception). Lastly, passive-avoidant leadership, the least effective and potentially counterproductive style, encompasses passive management by exception, indicating the leader to only intervene when things have already gone awry, and *laissez-faire* leadership, i.e., leader withdrawal and avoidance of responsibilities (Bass, 1985). The full range leadership model, however, not only ranked these behaviors and styles according to their effectiveness but also in terms of their level of leader activity, with idealized influence indicating both the highest leader activity and effectiveness, while *laissez-faire* corresponds with the lowest activity and effectiveness. As extraversion describes individuals displaying highly active, energetic, and sociable behaviors (Costa and McCrae, 1992; Soto and John, 2017), we expect observers to infer an overall higher degree of extraversion from more active and thus effective leadership styles and behaviors. This matches with previous findings linking extraversion to mainly transformational (Zaccaro et al., 2018), but also transactional leadership behaviors (Bono and Judge, 2004), and stating charismatic leadership, which encompasses the idealized influence and inspirational motivation behaviors (e.g., Towler, 2003; Antonakis et al., 2016), to even constitute a contextualized form of extraversion and therefore being best representative of this characteristic (de Vries, 2012, 2018).

*Hypothesis 1*: Ratings of extraversion will increase in tandem with the increasing effectiveness of leadership styles and behaviors.

On the second level, we disassemble extraversion into two of its specific constituents, assertiveness and sociability (Costa and McCrae, 1992; Soto and John, 2017) which is of particular relevance to studying the trait theory of leadership (Bono and Judge, 2004; Do and Minbashian, 2014). These dimensions are often described as the abilities of *getting ahead* and *getting along* (Hogan and Holland, 2003). More concretely, assertive individuals are characterized by being dominant and decisive and exerting more influence on others, which, overall, makes them appear more prototypical for leaders (Costa and McCrae, 1992; Hu et al., 2019). Assertiveness was also identified as the main driver for extraversions’ effects on performance increases (Judge et al., 2013; Do and Minbashian, 2014; Pearsall and Ellis, 2016). Sociable or warm individuals, on the other hand, focus on friendliness and bonding to gain popularity, acceptance, and a large social network (Costa and McCrae, 1992; Hu et al., 2019), thus facilitating leadership emergence (DeRue and Ashford, 2010; Judge et al., 2013; Hu et al., 2019). This differentiation between assertiveness and sociability, incidentally, shows parallels with the fundamental dimensions of social cognition—competence and warmth (see Fiske et al., 2007)—and more specifically matches with the influence and affability dimensions stated to constitute everyday charisma (Tskhay et al., 2018), a factor shown to greatly affect leader behavior (Maran et al., 2019, 2020). Others focused on these facets before and collected meta-analytic evidence that the agentic dimension of extraversion (encompassing the assertiveness aspect) appears to be the main driver behind the associations between extraversion, transformational leadership, and ultimately performance (Judge et al., 2013; Pearsall and Ellis, 2016), while the affiliative dimension (corresponding with sociability and warmth), did not (Do and Minbashian, 2014). This may be due to the fact that the the main task of leaders is to influence others (van Vugt et al., 2008; van Vugt and Ronay, 2014; Antonakis et al., 2016). A task that is greatly benefitted by the former stated characteristics of assertiveness. Thus, as assertiveness appears as an attribute that directly facilitates leaders to succeed in their core task and thus makes them more effective, we conversely expect leadership behaviors associated with successful influence to elicit greater attributions of assertiveness. Sociability, on the other hand and as described above, is less directly related to the effectiveness aspect of leadership (Judge et al., 2013; Hu et al., 2019) and while it should affect leaders’ success via the pathway of facilitating their emergence, within our study we already posit the leader in a formal leader role and therefore expect no difference of sociability ratings based on the described leadership styles and behaviors in general, though we expect specific effects on certain behaviors as detailed below.

*Hypothesis 2*: Ratings of assertiveness will increase in tandem with the increasing effectiveness of leadership styles and behaviors.

Similar to the differentiation between assertiveness and sociability, transformational leadership behaviors can be clustered into two dimensions: leader-centered (or charismatic) and follower-centered (or rational-developmental) behaviors (Antonakis et al., 2016). The former, encompassing idealized influence and inspirational motivation, are based on leaders’ skills to motivate followers. Both of these behaviors place the leader in a dominant central role in which they exert influence on their followers, again emphasizing the importance of being assertive to more easily engage in these behaviors. Follower-centered behaviors, on the other hand, are comprised of intellectual stimulation and individualized consideration, which focus on enhancing follower performance through considering and developing their individual skills. Intellectual stimulation describes a leader’s effort to develop their followers’ abilities to critically assess their assumptions and come up with own solutions rather than providing them with a direction to follow (Bass, 1985). Individualized consideration entails showing sympathy with followers’ unique strengths and weaknesses and paying attention to their needs (Bass, 1985). In both cases leaders position themselves more strongly in the service of their followers and while these tactics are still effective, they are less dependent by the leader directing their followers’ but rather gaining their trust and sympathy through personal connection that is representative of a sociable personality. These distinct leadership approaches, that might even, to a degree, be incompatible (see Antonakis et al., 2016) should therefore correspond with perceptions of the more self-centered assertiveness and the more other-regarding sociability facet of extraversion, respectively.

*Hypothesis 3a*: Ratings of assertiveness will be higher for leader-centered than follower-centered transformational leadershipbehaviors.

*Hypothesis 3b*: Ratings of sociability will be lower for leader-centered than follower-centered transformational leadership behaviors.

## 3. Materials and methods

To test these hypotheses, we prepared descriptions of coordination challenges, each accompanied by a distinct behavioral reaction from an imaginary leader, corresponding with one of the eight leadership dimensions according to the full-range leadership model. We directly contacted German-speaking adults through personal address as well as connections to organizations in Austria, Germany, Liechtenstein, and Switzerland and provided them with a link to our online questionnaire. They were randomly assigned to rate a set of four leadership behaviors regarding perceived leader extraversion, assertiveness, and sociability. Participants not completing the full questionnaire were excluded from all analyses.

### 3.1. Sample

Our final sample consisted of 204 German-speaking participants (134 female, 67 male, 3 diverse), the majority (76.0%) being young adults (age < 30; median age: 20–24 years). At the time of our study, most were living in Austria (54.9%), followed by Germany (26.5%), Liechtenstein (11.3%), and Switzerland (5.9%). 52% of our sample consisted of students, while 42.6% were currently employed. Most participants were working in the health or social sector (14.7%), followed by the public, industry, and trade (13.7% each), information and consulting (13.2%), and banking and finance (10.3%) sector. 85.8% of participants had at least 1 year of job experience (median 3 to 7 years). All participants provided informed consent.

### 3.2. Measures

We collected ratings of leaders’ assertiveness and sociability as facets of extraversion using the corresponding subscales from the Big Five Inventory-2 (BFI-2) (Soto and John, 2017; German translation by Danner et al., 2016). Each facet is composed of four statements participants had to indicate their agreement with on a 5-point Likert scale (1 = *strongly disagree*, 5 = *strongly agree*). Cronbach’s alpha reliability was at *α* = 0.86 for the assertiveness, *α* = 0.87 for the sociability, and *α* = 0.92 for the composite extraversion scale.

### 3.3. Stimulus material

Our scenarios were based on the leadership style assessment questionnaire (LSA) (Pundt, 2017), a situational judgment test of leadership behaviors. This questionnaire provides participants with eight scenarios, each accompanied by eight leadership behaviors based on the full-range leadership model they or their leaders could engage in should they find themselves in this scenario. We selected two distinct behavioral responses for four of the scenarios each, as stimulus material (see Appendix).

### 3.4. Data analysis

To assess differences in participants’ perceptions based on the distinct leadership styles and behaviors, we computed univariate ANOVAs for the eight leadership behaviors and the three leadership styles by aggregating ratings for the respective scenarios. Transformational leadership encompassed four conditions that received a total of 408 ratings, while the other styles received 204 ratings each. We conducted Welch’s tests, as well as Games-Howell *post-hoc* tests for pairwise comparisons to mitigate the resulting unequal distribution of variances. For analyses of the eight leadership behaviors, we used the regular *F*-statistic, as well as Scheffé tests to account for alpha error accumulation. Additionally, to identify linear trends in the observer ratings corresponding with the increases in leadership effectiveness as postulated by the full-range leadership model, we computed polynomial linear contrasts. To evaluate hypotheses 3a and 3b, we further combined the idealized influence and inspirational motivation conditions as leader-centered and the inspirational motivation and individualized consideration conditions as follower-centered transformational leadership behaviors and conducted t-tests to compare the mean assertiveness and sociability ratings. All data analyses were computed using *SPSS* (Version 26).

## 4. Results

### 4.1. Extraversion

**Figure 1:**
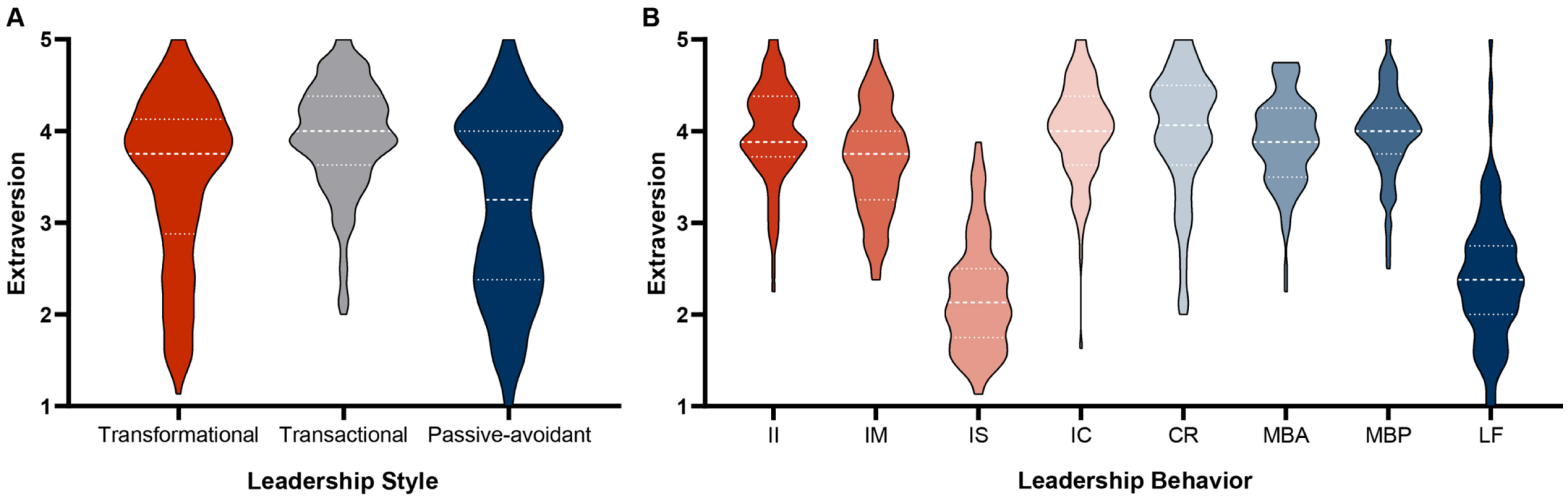
Violin plots depicting the extraversion ratings for the three leadership styles **(A)** and eight leadership behaviors **(B)**; II, idealized influence; IM, inspirational motivation; IS, intellectual stimulation; IC, individualized consideration; CR, contingent reward; MBA, active management by exception; MBP, passive management by exception; LF, *laissez-faire*. Dotted lines indicate the first and third quartile; the dashed line equals the median.

Firstly, we found extraversion ratings to differ depending on the three leadership styles (Welch’s *F*_2,457.26_ = 53.71, *p* < 0.001, *η_p_*^2^ = 0.09; see Table 1 for all means and standard errors of the ratings). Polynomial linear contrasts indicated a linear relationship between increasing leadership effectiveness and extraversion ratings (*t*_385.07_ = 3.40, *p* < 0.001) and we found ratings for both the transformational (*MD* = 0.28, *SE* = 0.08, *p*_G-H_ = 0.002; for an overview of the pairwise comparisons between leadership styles, see Table 2) and transactional (*MD* = 0.75, *SE* = 0.08, *p*_G-H_ < 0.001) leadership conditions to be higher than those for the passive-avoidant style. Yet, transformational leadership was rated lower than transactional (*MD* = −0.47, *SE* = 0.06, *p*_G-H_ < 0.001), and thus no clear linear relationship could be detected (see Figure 1A). We therefore refrain from interpreting the results of the polynomial linear contrast analyses as an actual linear association between the leadership behaviors’ effectiveness and participants’ ratings.

**Table 1:** Means (*M*) and standard errors (*SE*) of the extraversion, assertiveness, and sociability ratings for each leadership style (italic) and leadership behavior.

Leadership behavior		Extraversion		Assertiveness		Sociability	
	*N*	*M*	*SE*	*M*	*SE*	*M*	*SE*
*Transformational leadership*	408	3.46	0.05	3.44	0.05	3.48	0.05
Idealized influence	102	3.97	0.05	3.96	0.06	3.98	0.06
Inspirational motivation	102	3.67	0.06	3.83	0.07	3.50	0.06
Intellectual stimulation	102	2.20	0.06	2.16	0.07	2.25	0.06
Individualized consideration	102	4.00	0.05	3.81	0.07	4.18	0.06
*Transactional leadership*	204	3.93	0.04	3.75	0.05	4.11	0.05
Contingent reward	102	3.96	0.07	3.84	0.07	4.09	0.07
Active Mgmt. by exception	102	3.90	0.05	3.67	0.06	4.12	0.05
*Passive-avoidant leadership*	204	3.18	0.07	3.04	0.07	3.31	0.07
Passive Mgmt. by exception	102	3.96	0.05	3.77	0.06	4.15	0.06
Laissez-faire	102	2.40	0.07	2.31	0.08	2.48	0.07

**Table 2:** Mean differences between the extraversion, assertiveness, and sociability ratings for the three leadership styles.

	Extraversion		Assertiveness		Sociability		Transactional	Passive-avoidant	Transactional	Passive-avoidant	Transactional	Passive-avoidant
Transformational	−0.47***	0.28**	−0.31***	0.40***	−0.63***	0.16	[−0.62; −0.32]	[0.09; 0.48]	[−0.48; −0.15]	[0.20; 0.60]	[−0.78; −0.47]	[−0.04; 0.37]
Transactional		0.75***		0.71***		0.79***		[0.56; 0.94]		[0.51; 0.92]		[0.59; 1.00]

*p*-values were adjusted based on Games-Howell *post-hoc* tests.

Values in parentheses represent 95% confidence intervals of the mean differences.

**p < 0.01, ***p < 0.001.

Secondly, when it comes to the eight leadership behaviors, we found quite pronounced differences (*F*_7,808_ = 170.52, *p* < 0.001, *η_p_*^2^ = 0.60; Figure 1B); while polynomial contrasts again indicated a linear effect (*t*_808_ = 6.04, *p* < 0.001), we cannot really discern this linearity in our data due to *laissez-faire* being an expected and intellectual stimulation a rather unexpected outlier with both conditions differing significantly from all others (*MD* = [−1.79; −1.27], all *p*’s < 0.001; see Table 3). Intellectual stimulation was even rated lowest on extraversion, followed by *laissez-faire*, inspirational motivation, active then passive management by exception, contingent reward, idealized influence, and individualized consideration, thus further opposing Hypothesis 1.

**Table 3:** Mean differences between the extraversion ratings for the eight leadership behaviors.

	IM	IS	IC	CR	MBA	MBP	LF
II	0.31	1.77***	−0.02	0.01	0.07	0.01	1.58***
	[0.00; 0.62]	[1.46; 2.08]	[−0.33; 0.28]	[−0.30; 0.32]	[−0.23; 0.38]	[−0.30; 0.32]	[1.27; 1.89]
IM		1.46***	−0.33*	−0.30	−0.23	−0.30	1.27***
		[1.15; 1.77]	[−0.64; −0.02]	[−0.61; 0.01]	[−0.54; 0.07]	[−0.60; 0.01]	[0.96; 1.58]
IS			−1.79***	−1.76***	−1.69***	−1.76***	−0.19
			[−2.10; −1.49]	[−2.07; −1.45]	[−2.00; −1.39]	[−2.01; −1.45]	[−0.50; 0.12]
IC				0.03	0.10	0.04	1.60***
				[−0.27; 0.34]	[−0.21; 0.41]	[−0.27; 0.34]	[1.30; 1.91]
CR					0.06	0.00	1.57***
					[−0.24; 0.37]	[−0.31; 0.31]	[1.26; 1.88]
MBA						−0.06	1.50***
						[−0.37; 0.24]	[1.19; 1.81]
MBP							1.57***
							[1.26; 1.87]

II, idealized influence; IM, inspirational motivation; IS, intellectual stimulation; IC, individualized consideration; CR, contingent reward; MBA, active management by exception; MBP, passive management by exception; LF, *laissez-faire*.

*p*-values were adjusted based on Scheffé *post-hoc* tests.

Values in parentheses represent 95% confidence intervals of the mean differences.

*p < 0.05, ***p < 0.001.

### 4.2. Assertiveness

**Figure 2:**
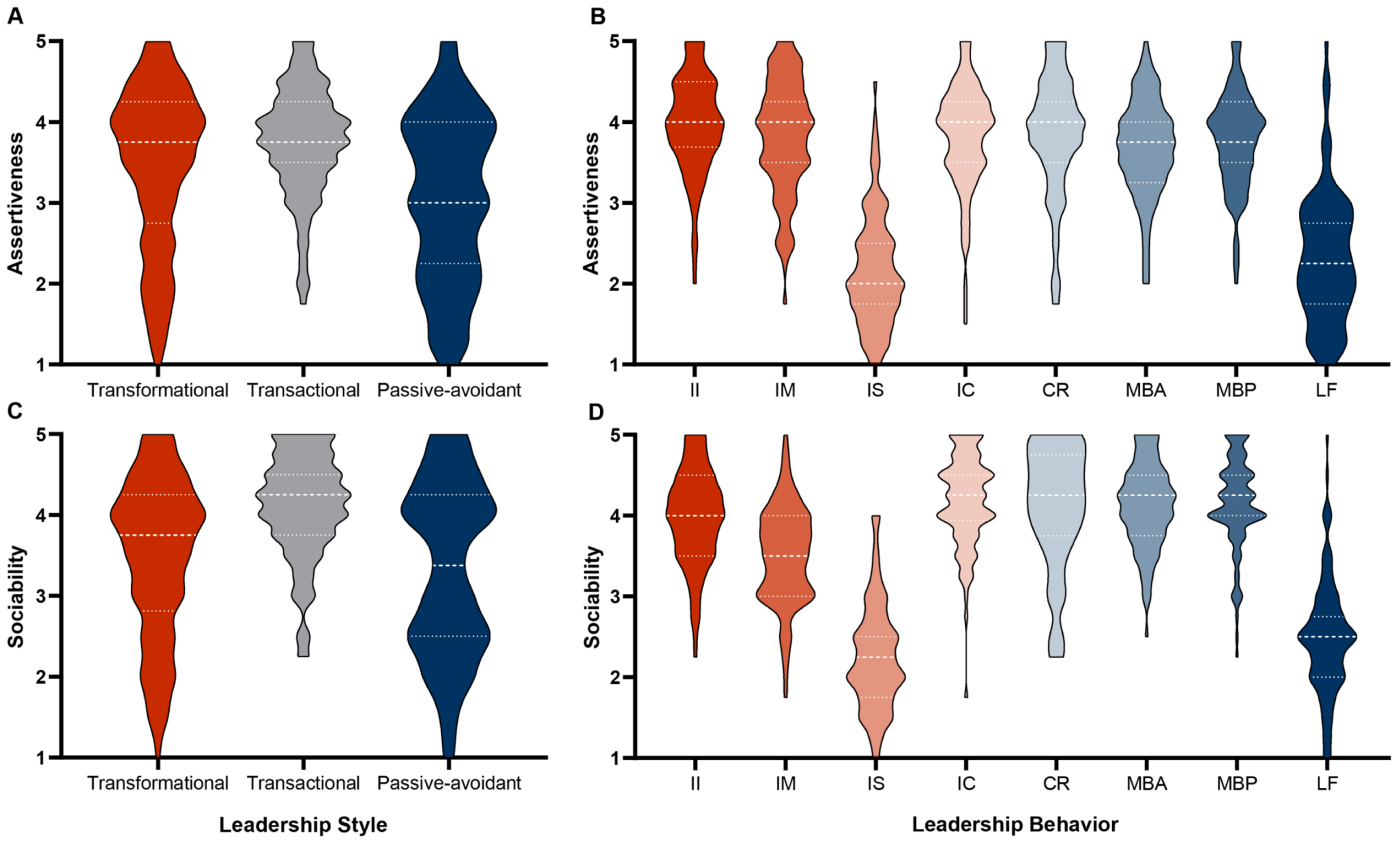
Violin plots depicting the assertiveness **(A,B)** and sociability **(C,D)** ratings for the three leadership styles **(A,C)** and eight leadership behaviors **(B,D)**, II, idealized influence; IM, inspirational motivation; IS, intellectual stimulation; IC, individualized consideration; CR, contingent reward; MBA, active management by exception; MBP, passive management by exception; LF, *laissez-faire*. Dotted lines indicate the first and third quartile; the dashed line equals the median.

Assertiveness ratings for the leadership styles showed a similar pattern of differences as did extraversion ratings (Welch’s *F*_2,458.42_ = 35.37, *p* < 0.001, *η_p_*^2^ = 0.07; see Table 1), with transformational (*MD* = 0.40, *SE* = 0.09, *p*_G-H_ < 0.001) and transactional leadership (*MD* = 0.71, *SE* = 0.09, *p*_G-H_ < 0.001) receiving higher ratings than passive-avoidant leadership, yet the most effective style getting rated lower than transactional leadership (*MD* = −0.31, *SE* = 0.07, *p*_G-H_ < 0.001; see Table 2). Therefore, the statistically significant polynomial contrasts (*t*_398.31_ = 4.60, *p* < 0.001) again did not relate to a detectable linear pattern (see Figure 2A).

Ratings based on the eight leadership behaviors were also strongly deviating from one another (*F*_7,808_ = 118.53, *p* < 0.001, *η_p_*^2^ = 0.51; see Table 1 and Figure 2B) and polynomial contrasts indicated a linear trend (*t*_808_ = 8.30, *p* < 0.001). However, as in the previous analysis, intellectual stimulation and *laissez-faire* were rated particularly low in assertiveness, while all other ratings remained on a roughly consistent level (see Table 4).

**Table 4:** Mean differences between the assertiveness ratings for the eight leadership behaviors.

	IM	IS	IC	CR	MBA	MBP	LF
II	0.13	1.80***	0.15	0.13	0.29	0.19	1.65***
	[−0.22; 0.50]	[1.44; 2.16]	[−0.21; 0.51]	[−0.23; 0.49]	[−0.07; 0.65]	[−0.17; 0.55]	[1.29; 2.01]
IM		1.66	0.01	−0.01	0.15	0.05	1.52***
		[1.30; 2.02]	[−0.35; 0.37]	[−0.37; 0.35]	[−0.21; 0.51]	[−0.31; 0.41]	[1.16; 1.88]
IS			−1.65***	−1.67***	−1.51***	−1.61***	−0.15
			[−2.01; −1.29]	[−2.03; −1.31]	[−1.87; −1.15]	[−1.97; −1.25]	[−0.51; 0.21]
IC				−0.02	0.14	0.04	1.50***
				[−0.38; 0.34]	[−0.22; 0.50]	[−0.32; 0.40]	[1.14; 1.86]
CR					0.16	0.06	1.53***
					[−0.20; 0.52]	[−0.30; 0.42]	[1.17; 1.89]
MBA						−0.10	1.37***
						[−0.46; 0.26]	[1.01; 1.73]
MBP							1.47***
							[1.11; 1.83]

II, idealized influence; IM, inspirational motivation; IS, intellectual stimulation; IC, individualized consideration; CR, contingent reward; MBA, active management by exception; MBP, passive management by exception; LF, *laissez-faire*.

*p*-values were adjusted based on Scheffé *post-hoc* tests.

Values in parentheses represent 95% confidence intervals of the mean differences.

***p < 0.001.

### 4.3. Sociability

Though we again could detect differences in ratings across the three higher order conditions (Welch’s *F*_2,453.76_ = 64.24, *p* < 0.001, *η_p_*^2^ = 0.10; see Table 1 and Figure 2C), these again did not correspond with our predictions, as transformational leadership was again rated lower than transactional leadership (*MD* = −0.63, *SE* = 0.07, *p*_G-H_ < 0.001), and in this case did not substantially differ from passive-avoidant leadership (*MD* = 0.16, *SE* = 0.09, *p*_G-H_ = 0.149). Transactional leadership remained getting rated higher than passive-avoidant (*MD* = 0.79, *SE* = 0.09, *p*_G-H_ < 0.001; see Table 2). In this instance, polynomial contrasts did not indicate a linear trend (*t*_375.50_ = 1.87, *p* = 0.062).

Ratings for leadership behaviors again were deviating strongly (*F*_7,808_ = 162.56, *p* < 0.001, *η_p_*^2^ = 0.59; see Table 1 and Figure 2D), with a statistically significant linear trend (*t*_808_ = 2.19, *p* = 0.029) that could not be detected visually. Similar to our previous analyses, intellectual stimulation and *laissez-faire* were rated lowest, yet sociability ratings were also lower for inspirational motivation, which was thus located in between intellectual stimulation and *laissez-faire* on the one hand and the other leadership behaviors on the other (see Table 5).

**Table 5:** Mean differences between the sociability ratings for the eight leadership behaviors.

	IM	IS	IC	CR	MBA	MBP	LF
II	0.48***	1.74***	−0.20	−0.11	−0.14	−0.16	1.50***
	[0.15; 0.81]	[1.41; 2.07]	[−0.53; 0.13]	[−0.44; 0.22]	[−0.47; 0.19]	[−0.49; 0.17]	[1.17; 1.83]
IM		1.26***	−0.68***	−0.59***	−0.62***	−0.64***	1.02***
		[0.93; 1.59]	[−1.01; −0.35]	[−0.92; −0.26]	[−0.95; −0.29]	[−0.97; −0.31]	[0.69; 1.36]
IS			−1.94***	−1.85***	−1.88***	−1.90***	−0.24
			[−2.27; −1.61]	[−2.18; −1.52]	[−2.21; −1.55]	[−2.23; −1.57]	[−0.57; 0.10]
IC				0.09	0.06	0.03	1.70***
				[−0.24; 0.42]	[−0.27; 0.39]	[−0.30; 0.36]	[1.37; 2.03]
CR					−0.03	−0.06	1.61***
					[−0.36; 0.30]	[−0.39; 0.27]	[1.28; 1.94]
MBA						−0.02	1.64***
						[−0.36; 0.31]	[1.31; 1.97]
MBP							1.67***
							[1.34; 2.00]

II, idealized influence; IM, inspirational motivation; IS, intellectual stimulation; IC, individualized consideration; CR, contingent reward; MBA, active management by exception; MBP, passive management by exception; LF, *laissez-faire.*

*p*-values were adjusted based on Scheffé *post-hoc* tests.

Values in parentheses represent 95% confidence intervals of the mean differences.

***p < 0.001.

### 4.4. Leader- vs. follower-centered behaviors

Lastly, when comparing the ratings for leader- and follower-centered transformational leadership behaviors, respectively, we found significant differences regarding assertiveness (*MD* = 0.91, *SE* = 0.09, *t*_337.51_ = 10.29, *p* < 0.001) and sociability ratings (*MD* = 0.53, *SE* = 0.09, *t*_319.68_ = 5.76, *p* < 0.001). In both cases, ratings were higher for the leader-centered behaviors. This, however, is attributable to the notably low ratings for intellectual stimulation, as, when comparing only individualized consideration to the leader-centered behaviors, we found sociability ratings to be higher in the former condition (*MD* = −0.44, *SE* = 0.08, *t*_304_ = −5.80, *p* < 0.001), while assertiveness ratings did not differ significantly (*MD* = 0.08, *SE* = 0.08, *t*_304_ = 1.01, *p* = 0.314), thus partly confirming *Hypothesis 3b*, yet opposing *Hypothesis 3a*.

## 5. Discussion

We set out to gather insights into the effects of leadership behaviors on observers’ perceptions of extraversion and its main facets of assertiveness and sociability. As previous research revealed, leaders appear to signal their extraversion by engaging in certain, particularly active and effective leadership behaviors (Bono and Judge, 2004; Zaccaro et al., 2018). When shifting the perspective from the leader as a sender to the follower as a perceiver of such signals, we expected ascriptions of extraversion based on observed leadership behaviors to increase in accordance with an increasing level of activity and effectiveness displayed by the leader. While our hypotheses could not be fully supported, we obtained some unexpected yet fascinating findings. In more detail, although they were indicated by polynomial linear contrasts, we did not find the expected linear trends of increased extraversion ratings corresponding to increased leadership effectiveness (*Hypothesis 1*), neither when analyzing on the level of leadership styles nor on the level of specific leadership behaviors. Further, we did not detect these trends for assertiveness ratings (*Hypothesis 2*). While we did not expect such a pattern for sociability, which was partly confirmed by our analyses, the results for these ratings still largely mirrored those for extraversion and assertiveness. The root cause for these findings was the universally low ratings for intellectual stimulation, which were at the same level as *laissez-faire* and strongly deviated from all other conditions. This unexpected deviation also affected our analyses of the differences in assertiveness and sociability ratings based on leader-centered and follower-centered transformational leadership behaviors. We expected higher ratings of assertiveness in the former (*Hypothesis 3a*) and higher ratings of sociability in the latter (*Hypothesis 3b*), which could not be confirmed. However, when only comparing the leader-centered behaviors to individualized consideration, sociability ratings were indeed higher for this behavior compared to the other two, thus partly coinciding with *Hypothesis 3b*. Though overall deviating from our expectations, our findings provide some thought-provoking insights and implications when searching for potential explanations for these effects. Firstly, all leadership behaviors employed as stimulus material, except for *laissez-faire*, detailed socially-oriented behaviors, yet of quite distinctive scopes. The high ratings for active and passive management by exception, and contingent reward may be explained by these conditions depicting frequent in-depth interactions between the leader and their followers (e.g., regularly meeting with them to control their performance). On the other hand, even though idealized influence and inspirational motivation depicted the leader interacting with their followers, these interactions were rather one-sided, with the leader either speaking about their values or motivating their followers, which might have resulted in lowered ratings. Yet, intellectual stimulation was rated as low in extraversion and its facets, despite depicting the leader interacting with their employees in the form of a shared discussion. The aspect of encouraging the followers to participate by bringing in their own ideas definitely opposes the typically discussion-dominating aspect of assertiveness (Hu et al., 2019), yet sociability being affected that strongly is rather counterintuitive.

This brings us to our second point of consideration. Previous studies found the extraverted leadership advantage to potentially be reversed in proactive teams (Grant et al., 2011). Therefore, effective leadership behaviors aimed at stimulating follower proactivity and developing their skills might be associated with more introverted leaders. So, while the behavior itself not necessarily signals low extraversion, it might be more regularly exhibited by less extraverted leaders, and, based on the participants’ own experiences, they might have recognized this behavior as atypical for extraverts. Interestingly, the two leadership behaviors resulting in ratings of low extraversion in our study, intellectual stimulation and *laissez-faire*, are also the two dimensions showing the strongest kinship to a different leadership concept said to be particularly effective at supporting proactivity (Harris et al., 2014; Zhang and Bartol, 2017), namely empowering leadership. The former bears semblance to the development support dimension of empowering leadership though providing less autonomy (Amundsen and Martinsen, 2014), while the latter is at times mixed up with it (Wong and Giessner, 2018). Though empowering leadership was shown to be less strongly predictive of task performance, it provides incremental explanatory power to transformational leadership in regard to other essential organizational outcomes (Lee et al., 2018). Therefore, while our prediction of extraversion ratings to increase in tandem with the effectiveness of leadership tactics was not supported, this might be explainable with extraversion’s relationship with more traditional effective leadership behaviors, whereas other behaviors of similar effectiveness could elicit inferences of introversion.

Thirdly and lastly, the findings that the degree of communication shown by the leader in our scenario did not seem to be the driver behind trait ratings, as described above, actually are in line with other recent findings (Mitchell et al., 2022). These postulate that communication skill should be investigated as distinct from extraversion because it could be the actual determinant of leader emergence.

Therefore, though unexpected, our results can actually be integrated rather well into the existing literature and this study further serves as a wellspring for future research opportunities.

### 5.1. Implications

The unexpected findings of our study and the discussion for their potential causes are implicating relevant topics for future research to investigate and allow for speculations on their potential practical relevance for introverted leaders and their choice of leadership style. While previous research suggested introverts to be able to act extraverted to emerge more easily and even experience more positive affect while doing so (McNiel and Fleeson, 2006; Howell et al., 2017; Spark and O’Connor, 2021), this should still constitute a costly practice to engage in in the long term, as it counteracts their natural behavioral instincts (Moskowitz and Coté, 1995; Pickett et al., 2020). Though, it should be noted, that state enactments of conscientiousness appear to lead to long-term beneficial outcomes irrespective of one’s trait conscientiousness (Kuijpers et al., 2022), and mostly counterdispositional enactments of introversion were found to entail detrimental effects (Zelenski et al., 2012; Kuijpers et al., 2021; Spark and O’Connor, 2021). Still, there is a need to better understand which leader behaviors introverts can engage in naturally and at a low cost, that allow for their emergence and especially long-term effectiveness. Intellectual stimulation behavior appears to better match with an introverted personality profile and might thus be less costly to enact than leader-self-centered behaviors while also providing the right prerequisites, such as development- and autonomy-support for followers to resolve their own issues rather than being at the center of discussions and disputes.

In more detail, our study shows that while the more effective leadership styles indicated by the full range leadership model (Bass, 1985) might be more suitable for extraverted individuals to engage in, effectiveness should not be equated with extraversion. At least one of the effective transformational leadership styles, intellectual stimulation, was perceived as more characteristic for an introverted personality. This finding is in line with recent literature on the positive influence of introverted leaders on proactive teams (Grant et al., 2011) and lends itself as an impetus for research on the modern and still unconventional yet highly effective (e.g., Lee et al., 2018) empowering leadership style (Amundsen and Martinsen, 2014). While ethical leadership was brought up as contextualized honesty-humility, supportive leadership as contextualized agreeableness, task-oriented leadership as contextualized conscientiousness, and charismatic leadership contextualized extraversion (de Vries, 2012, 2018), our findings on the connection between intellectual stimulation and introversion might indicate supportive and developmental leader practices, such as empowering leadership, to be on the flipside and potentially exemplify contextualized introversion. Yet, the claim of charismatic leadership being a contextualized form of extraversion could not actually be supported by our study, as the charisma-associated leadership behaviors idealized influence and inspirational motivation were not rated higher in neither extraversion, nor assertiveness or sociability as compared to individualized consideration or the transformational leadership behaviors. This might be due to mismatches in self- and observer-perceptions of personality that are particularly likely to occur for extraversion ratings (De Raad et al., 2008; de Vries, 2012). Still, empowering leadership seems to be a quite promising candidate for an effective leadership style that better matches an introverted personality profile.

Another concept that requires attention in the light of our findings is a more recent addition to leadership literature and an extension for the full range leadership model: instrumental leadership (e.g., Antonakis and House, 2014). This leadership style is explicitly based on the proposition that “effective organizational leadership is not just about exercising influence on an interpersonal level” (Antonakis and House, 2014, p. 747). It encompasses practices such as monitoring an organizations’ internal and external environment, as well as charting strategic and task-related objectives, and even the most social component, providing performance feedback to followers, does not demand a particularly assertive and sociable personality (Antonakis and House, 2014). Thus, it might be a further effective leadership style that is more easily accessible for introverts.

Ultimately, while displaying state extraversion might still be essential to emerge as a leader (Spark and O’Connor, 2021), in the longer run, introverted leadership behaviors, such as intellectual stimulation and related practices, might prove an alternative and less costly pathway to success given the right organizational context. Still, our study only provided an initial indication of the suitability of certain effective leadership behaviors for introverted individuals and this topic in general should be studied more extensively in future studies.

### 5.2. Limitations and future research directions

Our study presented the first expedition into investigating the impact of leadership behaviors on personality perceptions and therefore encompasses multiple limitations but also promising avenues for future research. Firstly, our study focused on leadership behaviors according to the full-range leadership model and employed extraversion ratings as the outcome variable due to its prominence in the trait approach to leadership. In this, we could not fully account for the complexities of the potential interrelations between leadership and personality. On the one hand, as touched upon above, different effective leadership styles, such as empowering (e.g., Amundsen and Martinsen, 2014) and instrumental leadership (Antonakis and House, 2014) might reveal entirely different relations between the effectiveness of the behavior and ascribed extraversion. On the other hand, extraversion is the most consistent but not sole predictor of leader success. Other dimensions of personality, most recently agreeableness (Blake et al., 2022), alternative personality models (Zuckerman et al., 1993), as well as dark personality dimensions (e.g., Kaiser et al., 2015) have shown relations to leadership and thus lend themselves as future research objects.

Secondly, and related to the notion of alternative leadership conceptualizations, we based our stimulus material on validated descriptions of behaviors from the LSA (Pundt, 2017). Charismatic and transformational leadership are frequently defined and assessed in an effect-centric manner (Antonakis et al., 2016), therefore doing otherwise could have led to endogeneity issues with our data. Yet, the stimuli presented only singular snapshots of specific leader behaviors in specific contexts, a limitation that could be overcome by using more universal and comprehensive descriptions (e.g., Stock et al., 2022).

Lastly, to keep our study focused on the relationships of interest, we did not assess potentially confounding factors or rater characteristics. For example, while we did not specify the gender of the leader in our scenarios, they might still have been perceived as belonging to a particular gender, and therefore their actions might have been perceived through a different lens (Paustian-Underdahl et al., 2014; Kim et al., 2020). Additionally, participants’ personality can also affect judgments of the leader, as these are potentially shaped by similarity bias (Felfe and Schyns, 2010). Including these in future research might allow for more precise estimates.

### 5.3. Conclusion

With this study, we intended to shed further light on the relationship between leader effectiveness and extraversion perceptions. We conducted an experimental investigation, providing participants with descriptions of leadership behaviors varying in effectiveness as proclaimed by the full-range leadership model (Bass, 1985). Contrary to our expectations, yet well explainable, ratings of extraversion, assertiveness, and sociability based on these behaviors were not linked to their respective effectiveness, as transformational leadership behaviors were rated only moderately in comparison to transactional leadership. This surprising disparity was based on intellectual stimulation and *laissez-faire* jointly receiving the lowest ratings. As both share similarities with the concept of empowering leadership, a style that can be quite effective in its own right, these findings provide an impetus for future research exploring potential relationships between introversion and leadership success. Similar to openness gaining more prominence as an essential employee characteristic for the modern workplace (D’Zurilla et al., 2011; Silvia and Christensen, 2020; Maran et al., 2022), introversion might be on the rise as a non-neglectable predictor of leaders’ success in developing proactive and empowered employees.

## Data availability statement

The datasets presented in this study can be found in online repositories. The names of the repository/repositories and accession number(s) can be found: Open Science Framework (OSF): https://osf.io/8jy4u/.

## Ethics statement

Ethical review and approval was not required for the study on human participants in accordance with the local legislation and institutional requirements. The patients/participants provided their written informed consent to participate in this study.

## Author contributions

SL: conceptualization, methodology, validation, investigation, formal analysis, writing—original draft, writing—review and editing, visualization, and project administration. MF: validation, resources, writing—original draft, writing—review and editing, supervision, and project administration. All authors contributed to the article and approved the submitted version.

## Funding

This research was supported by the Science Fund of the Principality of Liechtenstein (FFF; project no. ent_21_9).

## Conflict of interest

The authors declare that the research was conducted in the absence of any commercial or financial relationships that could be construed as a potential conflict of interest.

## Publisher’s note

All claims expressed in this article are solely those of the authors and do not necessarily represent those of their affiliated organizations, or those of the publisher, the editors and the reviewers. Any product that may be evaluated in this article, or claim that may be made by its manufacturer, is not guaranteed or endorsed by the publisher.

## Appendix

We presented participants with scenarios and descriptions of leadership behaviors based on the Leadership Styles Assessment (LSA) (Pundt, 2017). Below we provide translations for each of the scenarios and the accompanying leadership behaviors.

**Scenario 1**

An employee has submitted a presentation to their manager, which this employee is supposed to present to a potential customer. However, the quality of the presentation deviates significantly from the manager’s expectations. For example, basic formal guidelines and graphic design standards have not been adhered to. The manager does not agree with this performance.

*Idealized influence behavior:* The manager now behaves as follows: They talk to the employee about how important it is for them to present themselves professionally to potential customers and clarify their professional criticism without questioning the employee’s competencies.

*Active management by exception behavior:* The manager now behaves as follows: They ask the employee to revise their presentation. From now on, the manager regularly checks this employee to see if they make any mistakes or deviate from the guidelines again.

**Scenario 2**

Another dispute has arisen between two employees. The two employees share an office. Now one of the involved employees approaches their manager and asks for support. They feel that their concentration is being disturbed by their colleague, for example by their frequent telephone calls.

*Inspirational motivation behavior:* The manager now behaves as follows: They motivate both employees by emphasizing that their cooperation will contribute to the success of the department. The manager demonstrates their trust in both of them to come to a constructive and reasonable solution together.

*Laissez-faire behavior:* The manager now behaves as follows: They do not see it as their responsibility to deal with the disputes between the two employees. The manager leaves it to the employees to solve this problem among themselves.

**Scenario 3**

An employee has been working in their department for 3 years, but their original project expired after 2 years. The manager has therefore assigned them new tasks. For several months now, the employee has been regularly coming to the office late and not staying longer than necessary. Progress on their current project is very slow.

*Individualized consideration behavior:* The manager now behaves as follows: They ask the employee in a personal conversation why they are currently making little progress in their project and offer to support the employee by providing targeted feedback to help them further shape the project.

*Passive management by exception behavior:* The manager now behaves as follows: They wait to see what direction the employee’s work will take over the next few months. The manager only intervenes when the employee is about to abandon the project and quit.

**Scenario 4**

A new employee from another country and with a different cultural background joined the department a few weeks ago. Repeatedly, their manager noticed that this new employee spends their breaks individually and is not involved in the professional exchange between the other colleagues in the department.

*Intellectual stimulation behavior:* The manager now behaves as follows: They discuss with the employees and the foreign colleague how the professional exchange can be improved and encourage them to contribute creative ideas, such as an exchange about the two work cultures.

*Contingent reward behavior:* The manager now behaves as follows: They clarify to the employees that they expect more efforts to integrate the new colleague. In return, the manager offers to finance a joint team meal where everyone can get to know each other better.

## References

[ref1] Amundsen S. Martinsen Ø. L. (2014). Empowering leadership: construct clarification, conceptualization, and validation of a new scale. Leadersh. Q. 25, 487–511. doi: 10.1016/j.leaqua.2013.11.009

[ref2] Antonakis J. Bastardoz N. Jacquart P. Shamir B. (2016). Charisma: an ill-defined and ill-measured gift. Annu. Rev. Organ. Psych. Organ. Behav. 3, 293–319. doi: 10.1146/annurev-orgpsych-041015-062305

[ref3] Antonakis J. House R. J. (2014). Instrumental leadership: measurement and extension of transformational-transactional leadership theory. Leadersh. Q. 25, 746–771. doi: 10.1016/j.leaqua.2014.04.005

[ref4] Barrick M. R. Mount M. K. (1991). The big five personality dimensions and job performance: a meta-analysis. Pers. Psychol. 44, 1–26. doi: 10.1111/j.1744-6570.1991.tb00688.x, PMID: 34687041

[ref5] Bass Bernard M. (1985). Leadership performance beyond expectations. New York, NY: Academic Press.

[ref6] Blake A. B. Luu V. H. Petrenko O. V. Gardner W. L. Moergen K. J. N. Ezerins M. E. (2022). Let’s agree about nice leaders: a literature review and meta-analysis of agreeableness and its relationship with leadership outcomes. Leadersh. Q. 33:101593. doi: 10.1016/j.leaqua.2021.101593

[ref7] Bono J. E. Judge T. A. (2004). Personality and transformational and transactional leadership: a meta-analysis. J. Appl. Psychol. 89, 901–910. doi: 10.1037/0021-9010.89.5.901, PMID: 15506869

[ref8] Connelly B. L. Trevis Certo S. Duane Ireland R. Reutzel C. R. (2011). Signaling theory: a review and assessment. J. Manag. 37, 39–67. doi: 10.1177/0149206310388419, PMID: 37443594

[ref9] Costa P. T. McCrae R. R. (1992). Four ways five factors are basic. Personal. Individ. Differ. 13, 653–665. doi: 10.1016/0191-8869(92)90236-I, PMID: 36419047

[ref10] D’Zurilla T. J. Maydeu-Olivares A. Gallardo-Pujol D. (2011). Predicting social problem solving using personality traits. Personal. Individ. Differ. 50, 142–147. doi: 10.1016/j.paid.2010.09.015, PMID: 37101639

[ref11] Danner D. Rammstedt B. Bluemke M. Treiber L. Berres S. Soto C. J.. (2016). Die Deutsche Version Des Big Five Inventory 2 (BFI-2). Mannheim: GESIS - Leibniz-Institut für Sozialwissenschaften.

[ref12] De Raad B. Sullot E. Barelds D. P. H. (2008). Which of the big five factors are in need of situational specification? Eur. J. Personal. 22, 269–289. doi: 10.1002/PER.668

[ref13] de Vries R. E. (2012). Personality predictors of leadership styles and the self-other agreement problem. Leadersh. Q. 23, 809–821. doi: 10.1016/j.leaqua.2012.03.002

[ref14] de Vries R. E. (2018). Three nightmare traits in leaders. Front. Psychol. 9:319902. doi: 10.3389/FPSYG.2018.00871 PMC5994701 29915552

[ref15] DeRue D. S. Ashford S. J. (2010). Who will lead and who will follow? A social process of leadership identity construction in organizations. Acad. Manag. Rev. 35, 627–647. doi: 10.5465/AMR.35.4.ZOK627

[ref16] Do M. H. Minbashian A. (2014). A meta-analytic examination of the effects of the agentic and affiliative aspects of extraversion on leadership outcomes. Leadersh. Q. 25, 1040–1053. doi: 10.1016/j.leaqua.2014.04.004

[ref18] Elitzur R. Gavious A. (2003). Contracting, signaling, and moral Hazard: a model of entrepreneurs, ‘angels,’ and venture capitalists. J. Bus. Ventur. 18, 709–725. doi: 10.1016/S0883-9026(03)00027-2

[ref19] Ensari N. Riggio R. E. Christian J. Carslaw G. (2011). Who emerges as a leader? Meta-analyses of individual differences as predictors of leadership emergence. Personal. Individ. Differ. 51, 532–536. doi: 10.1016/j.paid.2011.05.017

[ref20] Felfe J. Schyns B. (2010). Followers’ personality and the perception of transformational leadership: further evidence for the similarity hypothesis. Br. J. Manag. 21, 393–410. doi: 10.1111/J.1467-8551.2009.00649.X

[ref21] Fiske S. T. Cuddy A. J. C. Glick P. (2007). Universal dimensions of social cognition: warmth and competence. Trends Cogn. Sci. 11, 77–83. doi: 10.1016/j.tics.2006.11.005, PMID: 17188552

[ref22] Fleeson W. Gallagher P. (2009). The implications of big five standing for the distribution of trait manifestation in behavior: fifteen experience-sampling studies and a meta-analysis. J. Pers. Soc. Psychol. 97, 1097–1114. doi: 10.1037/a0016786, PMID: 19968421 PMC2791901

[ref23] Furnham A. (2008). Personality and intelligence at work: Exploring and explaining individual differences at work. London: Routledge. doi: 10.4324/9780203938911

[ref24] Grabo A. Spisak B. R. van Vugt M. (2017). Charisma as signal: an evolutionary perspective on charismatic leadership. Leadersh. Q. 28, 473–485. doi: 10.1016/j.leaqua.2017.05.001

[ref25] Grant A. M. Gino F. Hofmann D. A. (2011). Reversing the extraverted leadership advantage: the role of employee proactivity. Acad. Manag. J. 54, 528–550. doi: 10.5465/amj.2011.61968043

[ref26] Harris T. B. Li N. Boswell W. R. Zhang X. A. Xie Z. (2014). Getting what’s new from newcomers: empowering leadership, creativity, and adjustment in the socialization context. Pers. Psychol. 67, 567–604. doi: 10.1111/PEPS.12053

[ref27] Hogan J. Holland B. (2003). Using theory to evaluate personality and job-performance relations: a Socioanalytic perspective. J. Appl. Psychol. 88, 100–112. doi: 10.1037/0021-9010.88.1.100, PMID: 12675398

[ref28] Howell R. T. Ksendzova M. Nestingen E. Yerahian C. Iyer R. (2017). Your personality on a good day: how trait and state personality predict daily well-being. J. Res. Pers. 69, 250–263. doi: 10.1016/J.JRP.2016.08.001

[ref29] Hu J. Zhang Z. Jiang K. Chen W. (2019). Getting ahead, getting along, and getting prosocial: examining extraversion facets, peer reactions, and leadership emergence. J. Appl. Psychol. 104, 1369–1386. doi: 10.1037/apl0000413, PMID: 30998025

[ref30] Judge T. A. Bono J. E. Ilies R. Gerhardt M. W. (2002). Personality and leadership: a qualitative and quantitative review. J. Appl. Psychol. 87, 765–780. doi: 10.1037/0021-9010.87.4.765, PMID: 12184579

[ref31] Judge T. A. Rodell J. B. Klinger R. L. Simon L. S. Crawford E. R. (2013). Hierarchical representations of the five-factor model of personality in predicting job performance: integrating three organizing frameworks with two theoretical perspectives. J. Appl. Psychol. 98, 875–925. doi: 10.1037/a0033901, PMID: 24016206

[ref32] Kaiser R. B. LeBreton J. M. Hogan J. (2015). The dark side of personality and extreme leader behavior. Appl. Psychol. 64, 55–92. doi: 10.1111/apps.12024

[ref33] Kim J. Y. Hsu N. Newman D. A. Harms P. D. Wood D. (2020). Leadership perceptions, gender, and dominant personality: the role of normality evaluations. J. Res. Pers. 87:103984. doi: 10.1016/J.JRP.2020.103984

[ref34] Kuijpers E. Pickett J. Wille B. Hofmans J. (2021). Do you feel better when you behave more extraverted than you are? The relationship between cumulative Counterdispositional extraversion and positive feelings. Personal. Soc. Psychol. Bull. 48, 606–623. doi: 10.1177/01461672211015062 34056978

[ref35] Kuijpers E. Pickett J. Wille B. Hofmans J. (2022). Does it pay off to act conscientiously, both now and later? Examining concurrent, lagged, and cumulative effects of state conscientiousness. Eur. J. Personal.:089020702211247. doi: 10.1177/08902070221124705

[ref36] Lee A. Willis S. Tian A. W. (2018). Empowering leadership: a meta-analytic examination of incremental contribution, mediation, and moderation. J. Organ. Behav. 39, 306–325. doi: 10.1002/job.2220

[ref37] Maran T. K. Furtner M. R. Liegl S. Kraus S. Sachse P. (2019). In the eye of a leader: eye-directed gazing shapes perceptions of leaders’ Charisma. Leadersh. Q. 30:101337. doi: 10.1016/j.leaqua.2019.101337

[ref38] Maran T. K. Liegl S. Davila A. Moder S. Kraus S. Mahto R. V. (2022). Who fits into the digital workplace? Mapping digital self-efficacy and agility onto psychological traits. Technol. Forecast. Soc. Chang. 175:121352. doi: 10.1016/J.TECHFORE.2021.121352

[ref39] Maran T. K. Moder S. Furtner M. R. Ravet-Brown T. Liegl S. (2020). From self-report to behavior: mapping Charisma onto naturalistic gaze patterns. Personal. Individ. Differ. 152:109562. doi: 10.1016/j.paid.2019.109562

[ref40] McNiel J. M. Fleeson W. (2006). The causal effects of extraversion on positive affect and neuroticism on negative affect: manipulating state extraversion and state neuroticism in an experimental approach. J. Res. Pers. 40, 529–550. doi: 10.1016/j.jrp.2005.05.003

[ref41] Mitchell T. James Lemoine G. Lee D. (2022). Inclined but less skilled? Disentangling extraversion, communication skill, and leadership emergence. J. Appl. Psychol. 107, 1524–1542. doi: 10.1037/apl0000962, PMID: 34618518

[ref42] Moskowitz D. S. Coté S. (1995). Do interpersonal traits predict affect? A comparison of three models. J. Pers. Soc. Psychol. 69, 915–924. doi: 10.1037/0022-3514.69.5.915, PMID: 21981891

[ref43] Paustian-Underdahl S. C. Walker L. S. Woehr D. J. (2014). Gender and perceptions of leadership effectiveness: a meta-analysis of contextual moderators. J. Appl. Psychol. 99, 1129–1145. doi: 10.1037/a0036751, PMID: 24773399

[ref44] Pearsall M. J. Ellis A. P. J. (2016). The effects of critical team member assertiveness on team performance and satisfaction. J. Manag. 32, 575–594. doi: 10.1177/0149206306289099

[ref45] Pickett J. Hofmans J. Debusscher J. De Fruyt F. (2020). Counterdispositional conscientiousness and wellbeing: how does acting out of character relate to positive and negative affect at work? J. Happiness Stud. 21, 1463–1485. doi: 10.1007/s10902-019-00139-1

[ref46] Pundt A. (2017). Leadership style assessment (LSA). Zeitschrift Arbeits 61, 152–158. doi: 10.1026/0932-4089/a000245

[ref47] Silvia P. J. Christensen A. P. (2020). Looking up at the curious personality: individual differences in curiosity and openness to experience. Curr. Opin. Behav. Sci. 35, 1–6. doi: 10.1016/J.COBEHA.2020.05.013

[ref48] Soto C. J. John O. P. (2017). The next big five inventory (BFI-2): developing and assessing a hierarchical model with 15 facets to enhance bandwidth, fidelity, and predictive power. J. Pers. Soc. Psychol. 113, 117–143. doi: 10.1037/pspp0000096, PMID: 27055049

[ref49] Spark A. O’Connor P. J. (2021). State extraversion and emergent leadership: do introverts emerge as leaders when they act like extraverts? Leadersh. Q. 32:101474. doi: 10.1016/j.leaqua.2020.101474

[ref50] Spark A. O’Connor P. J. Jimmieson N. L. Niessen C. (2022). Is the transition to formal leadership caused by trait extraversion? A counterfactual hazard analysis using two large panel datasets. Leadersh. Q. 33:101565. doi: 10.1016/j.leaqua.2021.101565

[ref51] Spence M. (1973). Job market signaling. Q. J. Econ. 87:355. doi: 10.2307/1882010, PMID: 37238540

[ref52] Spisak B. R. Homan A. C. Grabo A. Van Vugt M. (2012). Facing the situation: testing a biosocial contingency model of leadership in intergroup relations using masculine and feminine faces. Leadersh. Q. 23, 273–280. doi: 10.1016/j.leaqua.2011.08.006

[ref53] Stiglitz J. E. (2000). The contributions of the economics of information to twentieth century economics. Q. J. Econ. 115, 1441–1478. doi: 10.1162/003355300555015, PMID: 37139226

[ref54] Stock G. Banks G. C. Voss E. N. Tonidandel S. Woznyj H. (2022). Putting leader (follower) behavior Back into transformational leadership: a theoretical and empirical course correction. Leadersh. Q.:101632. doi: 10.1016/J.LEAQUA.2022.101632

[ref55] Thorndike E. L. (1920). A constant error in psychological ratings. J. Appl. Psychol. 4, 25–29. doi: 10.1037/h0071663, PMID: 35183242

[ref56] Tooby J. Cosmides L. (2008). “The evolutionary psychology of the emotions and their relationship to internal regulatory variables” in Handbook of emotions. eds. Lewis M. Haviland-Jones J. M. Barrett L. F. (New York, NY: The Guilford Press), 114–137.

[ref57] Towler A. J. (2003). Effects of charismatic influence training on attitudes, behavior, and performance. Pers. Psychol. 56, 363–381. doi: 10.1111/j.1744-6570.2003.tb00154.x

[ref58] Tskhay K. O. Zhu R. Zou C. Rule N. O. (2018). Charisma in everyday life: conceptualization and validation of the general Charisma inventory. J. Pers. Soc. Psychol. 114, 131–152. doi: 10.1037/pspp0000159, PMID: 28737418

[ref59] van Vugt M. Hogan R. Kaiser R. B. (2008). Leadership, followership, and evolution: some lessons from the past. Am. Psychol. 63, 182–196. doi: 10.1037/0003-066X.63.3.182, PMID: 18377108

[ref60] van Vugt M. Ronay R. (2014). The evolutionary psychology of leadership. Organ. Psychol. Rev. 4, 74–95. doi: 10.1177/2041386613493635

[ref61] Wilmot M. P. Ones D. S. (2019). A century of research on conscientiousness at work. Proc. Natl. Acad. Sci. U. S. A. 116, 23004–23010. doi: 10.1073/pnas.1908430116 31666330 PMC6859351

[ref62] Wilmot M. P. Wanberg C. R. Kammeyer-Mueller J. D. Ones D. S. (2019). Extraversion advantages at work: a quantitative review and synthesis of the Meta-analytic evidence. J. Appl. Psychol. 104, 1447–1470. doi: 10.1037/apl0000415, PMID: 31120263

[ref63] Wong S. I. Giessner S. R. (2018). The thin line between empowering and laissez-faire leadership: an expectancy-match perspective. J. Manag. 44, 757–783. doi: 10.1177/0149206315574597

[ref64] Zaccaro S. J. Green J. P. Dubrow S. Kolze M. J. (2018). Leader individual differences, situational parameters, and leadership outcomes: a comprehensive review and integration. Leadersh. Q. 29, 2–43. doi: 10.1016/j.leaqua.2017.10.003

[ref65] Zelenski J. M. Santoro M. S. Whelan D. C. (2012). Would introverts be better off if they acted more like extraverts? Exploring emotional and cognitive consequences of counterdispositional behavior. Emotion 12, 290–303. doi: 10.1037/a0025169, PMID: 21859197

[ref66] Zhang X. Bartol K. M. (2017). Linking empowering leadership and employee creativity: the influence of psychological empowerment, intrinsic motivation, and creative process engagement. Acad. Manag. J. 53, 107–128. doi: 10.5465/AMJ.2010.48037118

[ref67] Zuckerman M. Michael Kuhlman D. Joireman J. Teta P. Kraft M. (1993). A comparison of three structural models for personality: the big three, the big five, and the alternative five. J. Pers. Soc. Psychol. 65, 757–768. doi: 10.1037/0022-3514.65.4.757

